# Association of zinc deficiency and risk of new-onset dementia: a retrospective cohort study

**DOI:** 10.3389/fnut.2025.1666887

**Published:** 2025-11-04

**Authors:** Sheng-Han Huang, Hsiu-Lan Weng, Kuo-Chuan Hung, Chun-Ning Ho, Wen-Wen Tsai, Yi-Chen Lai, Jheng-Yan Wu, I-Wen Chen

**Affiliations:** ^1^Department of Anesthesiology, Chi Mei Medical Center, Tainan, Taiwan; ^2^Department of Anesthesiology, E-Da Hospital, I-Shou University, Kaohsiung, Taiwan; ^3^Department of Neurology, Chi Mei Medical Center, Tainan, Taiwan; ^4^Department of Nutrition, Chi Mei Medical Center, Tainan, Taiwan; ^5^Department of Anesthesiology, Chi Mei Medical Center, Liouying, Tainan, Taiwan

**Keywords:** zinc deficiency, dementia, cognitive decline, risk factors, cohort study, micronutrients

## Abstract

**Background:**

Zinc deficiency may contribute to cognitive decline through neuroinflammation and synaptic dysfunction; however, epidemiological evidence linking zinc deficiency to incident dementia remains limited. We investigated whether zinc deficiency is associated with an increased risk of new-onset dementia in a large healthcare population.

**Methods:**

We conducted a retrospective cohort study using the TriNetX Research Network and analyzed adults aged ≥50 years who underwent serum zinc testing between January 2010 and December 2023. Patients were stratified by zinc levels: deficiency (<70 μg/ml) vs. normal (70–120 μg/ml). After excluding those with pre-existing cognitive impairment or conditions affecting zinc metabolism, we performed 1:1 propensity score matching based on demographics, comorbidities, medications, and laboratory parameters. The primary outcome was new-onset dementia within 3 years. Cognitive impairment was assessed as an additional outcome, whereas pneumonia was included as a positive control outcome to validate the study's analytic approach.

**Results:**

After propensity score matching, 34,249 patients were included in each group. Zinc deficiency was associated with a 34% increased dementia risk (adjusted HR 1.34, 95% CI 1.17–1.53, *p* < 0.001) and 72% increased pneumonia risk (adjusted HR 1.72, 95% CI 1.63–1.81, *p* < 0.001). Cognitive impairment showed no significant association in the primary analysis (adjusted HR 1.08, 95% CI 0.92–1.28, *p* = 0.339) but became significant when the analysis was restricted to the pre-pandemic period (2010–2019, adjusted HR 1.38, 95% CI 1.11–1.72, *p* = 0.004). A clear dose-response relationship emerged when comparing both mild-to-moderate deficiency (50–70 μg/ml, adjusted HR 1.26, 95% CI 1.10–1.46) and severe deficiency (<50 μg/ml, adjusted HR 1.71, 95% CI 1.36–2.16) against normal zinc levels.

**Conclusion:**

Zinc deficiency represents an independent, modifiable risk factor for new-onset dementia with a clear dose-response relationship. These findings support the consideration of zinc status assessment and optimization in dementia prevention strategies. Future randomized controlled trials are warranted to establish causality and determine optimal intervention protocols.

## 1 Introduction

Dementia represents one of the most pressing global health challenges of the 21st century, with its prevalence continuing to rise worldwide (1–3). This progressive neurodegenerative condition has devastating consequences on patients, families, and healthcare systems, creating an enormous burden on society (4–6). Despite decades of intensive research, current therapeutic options remain largely symptomatic, with no disease-modifying treatments proven to halt or reverse cognitive decline (7, 8). Given the limited efficacy of available interventions and the absence of curative therapies, prevention has emerged as the most viable strategy for addressing this growing crisis. Research has increasingly focused on identifying modifiable risk factors across multiple domains, including cardiovascular health, lifestyle behaviors, environmental exposure, and nutritional status (9–11). Among these potential targets, nutritional deficiencies represent particularly promising intervention opportunities because they are both preventable and treatable (12–14), offering accessible pathways for reducing the risk of cognitive decline in diverse populations.

Zinc, an essential trace element abundantly present in brain tissue, plays a fundamental role in maintaining neurological health through multiple interconnected mechanisms (15–17). This vital micronutrient supports synaptic transmission, neuronal development, and cellular protection against oxidative stress, which accelerates brain aging (15–18). Zinc deficiency has been shown to compromise blood-brain barrier integrity, promote neuroinflammation, and disrupt neurotransmitter metabolism (19–21), all of which are pathological hallmarks of dementia progression. Animal and observational studies have increasingly highlighted a potential association between reduced serum zinc levels and cognitive decline (22–24), while experimental evidence demonstrates that zinc supplementation may improve memory function in selected human populations (25–28). These converging lines of evidence suggest that maintaining an adequate zinc status may represent a modifiable factor in preserving cognitive function throughout aging.

However, robust epidemiological evidence establishing zinc deficiency as an independent risk factor for incident dementia remains limited. Previous investigations have been hampered by relatively small sample sizes, inadequate follow-up durations, and insufficient controls for important confounding variables that could influence both zinc status and dementia risk (24, 29). Therefore, the clinical significance of zinc deficiency as a potentially modifiable risk factor for dementia prevention has not been definitively established in large, diverse healthcare populations. To address this critical knowledge gap, we conducted a retrospective cohort study utilizing a large, multicenter database to investigate whether zinc deficiency is independently associated with an increased risk of new-onset dementia.

## 2 Methods

### 2.1 Study design and data source

We performed a retrospective cohort analysis using data retrieved from the TriNetX Research Network. The TriNetX Research Network is a global, federated, real-time platform integrating de-identified electronic health records (EHRs) from over 100 healthcare organizations, primarily large academic centers, community hospitals, and outpatient clinics across North America, Europe, and Asia. Available data include demographic characteristics, diagnostic codes, procedures, laboratory results, and prescribed medications, representing a broad cross-section of patients rather than a single occupational or socio-demographic group. All data accessed through TriNetX are de-identified in compliance with the Health Insurance Portability and Accountability Act (HIPAA) Privacy Rule and do not include individually identifiable information. The TriNetX network has served as a robust data source for numerous studies in various clinical areas, including perioperative care, chronic illness management, and evaluation of health disparities (30–33). As this study used only de-identified retrospective data, the requirement for informed consent was waived. The study protocol was reviewed and approved by the Institutional Review Board of Chi Mei Medical Center (IRB number: 11310-E04).

### 2.2 Study population

**Figure 1:**
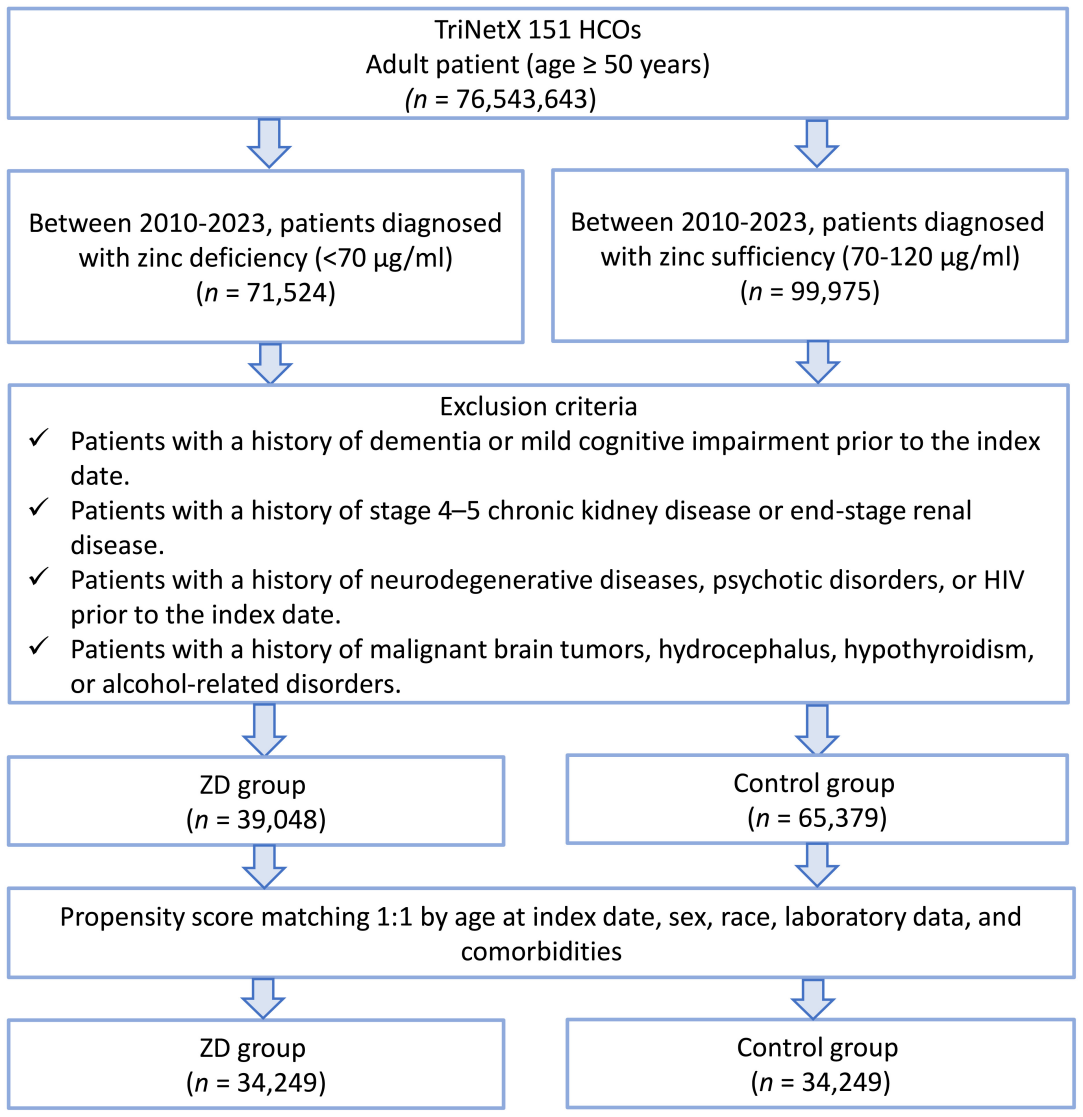
Patient selection flowchart from the TriNetX database. The flowchart illustrates the systematic exclusion process applied to identify eligible patients with zinc deficiency (ZD) and zinc sufficiency (control group). HCOs, healthcare organizations.

This study was a retrospective cohort analysis in which temporality was maintained (serum zinc measurement preceded dementia onset), but the retrospective nature arose from reliance on pre-existing electronic health records in the TriNetX network rather than active prospective follow-up. We identified adults aged ≥50 years who underwent serum zinc level testing between January 1, 2010, and December 31, 2023. Based on their zinc concentrations at the time of testing, individuals were stratified into two groups: those with serum zinc levels below 70 μg/ml were classified as having zinc deficiency, while those with levels ranging from 70 to 120 μg/ml were considered to have normal zinc status. The date of serum zinc measurement was designated as the index date for follow-up. A total of 76,543,643 adult patients (aged ≥ 50 years) from 151 healthcare organizations (HCOs) within the TriNetX network were initially screened (Figure 1). Of these, only 0.2% had serum zinc measurements available, which defined the eligible cohort for further analyses.

To ensure the validity of our cohort and minimize confounding, we implemented several pre-specified exclusion criteria. First, patients with any prior diagnosis of mild cognitive impairment (ICD-10: G31.84) and dementia, including vascular dementia (F01), unspecified dementia (F03), or dementia in other diseases (F02), before the index date (the date of serum zinc measurement) were excluded to focus on new-onset cases and avoid misclassification of pre-existing cognitive impairment. Second, we excluded patients with stage 4 or 5 chronic kidney disease or end-stage renal disease, given that advanced renal dysfunction can significantly alter zinc homeostasis and independently increase the risk of cognitive decline (34). Third, we excluded individuals with pre-existing conditions strongly associated with neurodegeneration, altered cognition, or severe systemic illnesses, including schizophrenia and other psychotic disorders (F20–F29), multiple sclerosis (G35), Parkinson's disease (G20), Huntington's disease (G10), HIV infection (B20), other degenerative diseases of the nervous system (G31), malignant brain tumors (C71), hydrocephalus (G91), hypothyroidism (E03), and alcohol-related disorders (F10).

### 2.3 Data collection and propensity score matching

Serum zinc concentrations were measured by the clinical laboratories of each participating healthcare organization as part of routine patient care. However, the analytical methods used for zinc quantification (such as atomic absorption spectrophotometry or inductively coupled plasma mass spectrometry) are not specified in the TriNetX database. All results were extracted as recorded in the electronic health records, and each institution followed its own standard operating procedures and quality control protocols.

Baseline demographic and clinical data were collected from the 3-year period preceding the index date for each patient. To reduce potential confounding and enhance group comparability, we performed 1:1 propensity score matching using a greedy nearest-neighbor algorithm without replacement. Although this approach resulted in a reduced sample size, it allowed for more rigorous control of measured confounders and improved internal validity. The matching model included demographic factors, such as age, sex, race, body mass index (BMI), comorbidities, and laboratory data. Medication exposure known to impact cognitive function or dementia risk was also incorporated into the matching process. These include benzodiazepine derivative sedatives/hypnotics, antidepressants, antiepileptic drugs, antilipemic agents, anticholinergics, GLP-1 receptor agonists, and SGLT2 inhibitors. To further minimize bias from the underlying nutritional status, we also matched for documented diagnoses of malnutrition, other nutritional deficiencies, and zinc supplementation. Medication use was defined as “ever use” if a prescription was documented within the 3-year pre-index window. Laboratory variables were incorporated into the propensity score model as categorical measures based on clinically relevant cutoffs.

### 2.4 Outcome definitions

The primary outcome of this study was the incidence of newly diagnosed dementia (ICD-10: F01, F02, F03) within the 3-year follow-up period after the index date. The secondary outcome was the risk of cognitive impairment. Cognitive impairment was defined as a new diagnosis of mild cognitive impairment (ICD-10: G31.84) in the electronic health records during the follow-up period. These codes are routinely entered by treating clinicians or coding professionals for clinical documentation and billing purposes and represent the standard method of outcome ascertainment in large EHR- and claims-based epidemiological studies. To reduce the misclassification of pre-existing cases, we applied a 3-month washout period after the index date and excluded events occurring during this interval. This minimized the risk of including undiagnosed conditions at the baseline.

In addition to evaluating the dementia incidence at the 3-year mark, we also assessed outcomes at 5-year intervals to explore the temporal consistency of zinc-related dementia risk over longer follow-up durations. Follow-up was defined as the period from the index date (serum zinc measurement) until the earliest occurrence of dementia, death, loss to follow-up, or the end of the observation window (3 or 5 years, depending on the analysis). For subjects whose index date was close to the end of data availability (December 31, 2023), follow-up may have been < 3 years. This variation in follow-up time was accounted for using time-to-event (survival) analysis. Although the follow-up duration of 3 to 5 years may be considered relatively short for dementia research, this approach was chosen to minimize exposure misclassification, as serum zinc concentrations are likely to change substantially over longer periods. Shorter follow-up may reduce the absolute number of dementia cases identified; however, both groups were followed for the same time window, ensuring comparability of risk estimates across exposure categories.

In the current study, pneumonia was deliberately included as a “positive control” outcome, based on established literature linking zinc deficiency to impaired immune response and increased infection risk (35). This methodological strategy is recommended in large-scale electronic health record-based studies to validate the reliability of the analytic approach or data integrity (36). If the association between zinc deficiency and pneumonia risk was not observed, it would cast doubt on the analytic approach or data integrity.

### 2.5 Sensitivity analysis

To evaluate the robustness of our primary findings and account for potential temporal confounding, we conducted a pre-specified sensitivity analysis that restricted the study period to patients with serum zinc level testing between January 1, 2010, and December 31, 2019. This approach was designed to exclude the potential influence of the COVID-19 pandemic on healthcare utilization patterns, diagnostic practices, and patient outcomes that may have emerged from 2020 onwards. The pandemic introduced significant disruptions in routine medical care, delayed preventive screenings, and altered clinical decision-making processes that could potentially confound the relationship between zinc deficiency and dementia risk.

### 2.6 Dose–response relationship

To investigate the potential dose-response relationship between serum zinc levels and dementia risk, we subdivided patients with zinc deficiency into two severity categories. Zinc deficiency was classified as mild-to-moderate (50–70 μg/ml) or severe (< 50 μg/ml) based on previously published literature (37). We then conducted two separate comparisons against the same control group (70–120 μg/ml): (1) mild-to-moderate zinc deficiency vs. control group and (2) severe zinc deficiency vs. control group. This approach allows the assessment of whether more severe zinc deficiency demonstrates a stronger association with dementia risk, thereby supporting a dose-response relationship.

### 2.7 Statistical analysis

Descriptive statistics were used to summarize the baseline characteristics. Continuous variables were reported as means with standard deviations, and categorical variables were expressed as counts and proportions. The TriNetX platform provides only aggregate summary statistics for continuous variables, such as mean and standard deviation, but does not allow access to individual-level raw data for formal normality testing or alternative data presentation (e.g., median value and interquartile range). Therefore, all continuous variables were reported as means with standard deviations, consistent with the data available from the platform. To evaluate the quality of propensity score matching, we calculated standardized mean differences (SMDs), with values below 0.1 considered indicative of adequate covariate balance. Propensity score distributions were also visually inspected to confirm overlap between the groups.

Cox proportional hazards models were applied to estimate unadjusted and adjusted hazard ratios (HRs) with corresponding 95% confidence intervals, accounting for censoring and varying risk over time. The proportional hazards (PH) assumption for Cox regression models was evaluated using Schoenfeld residuals. The following covariates were included in the multivariable Cox models: male sex, essential (primary) hypertension, overweight and obesity, diabetes mellitus, cerebrovascular diseases, malnutrition, anemia, liver disease, ischemic heart diseases, age at the index date, dyslipidemia, chronic kidney disease, and heart failure. To explore potential effect modification by demographic factors, we conducted pre-specified subgroup analyses stratified by sex (male vs. female) and age group (50–75 years vs. >75 years). All statistical analyses were performed using integrated tools within the TriNetX analytics platform, and a two-tailed *p*-value of < 0.05 was considered statistically significant throughout.

## 3 Results

### 3.1 Patient selection and baseline characteristics

**Figure 2:**
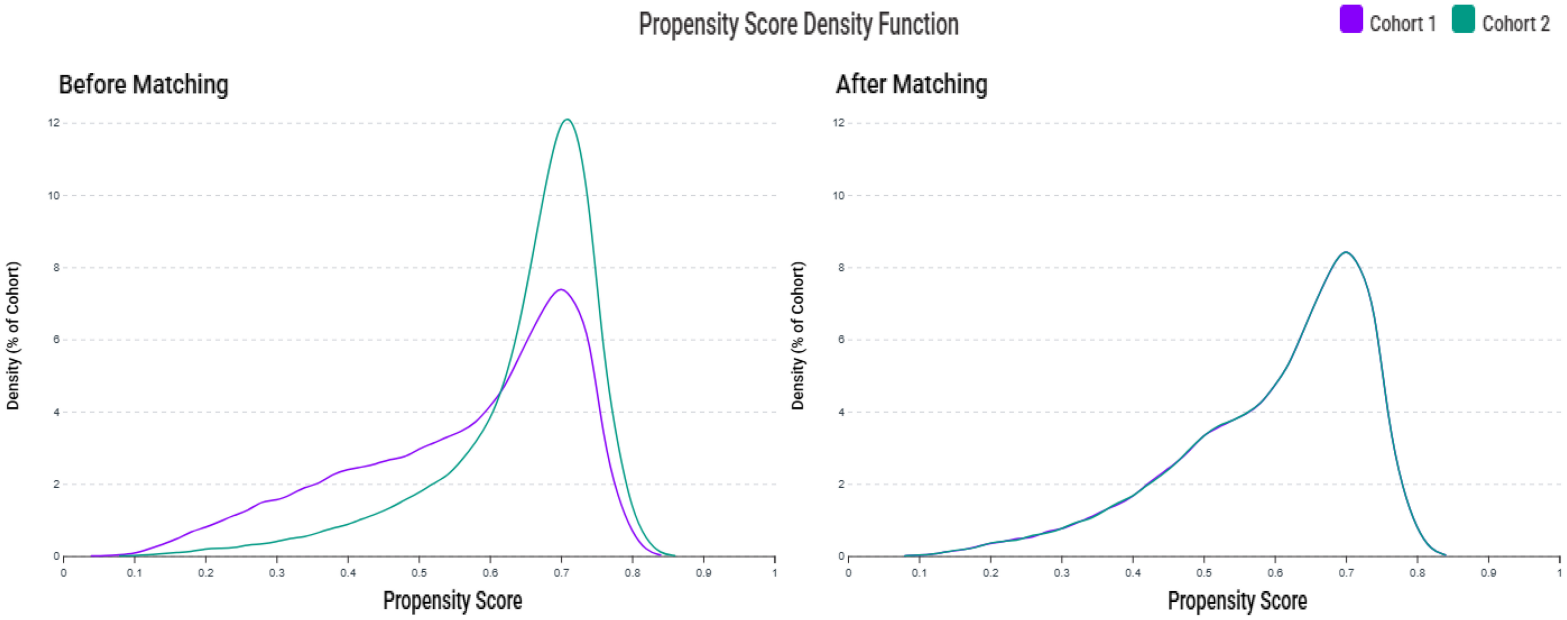
Propensity score density distributions before and after matching. The left panel shows the distribution of propensity scores between the zinc deficiency group (Cohort 1, purple) and the control group (Cohort 2, green) before matching, indicating a noticeable imbalance. The right panel shows improved overlap and covariate balance after 1:1 propensity score matching using age, sex, race, laboratory data, and comorbidities.

A total of 76,543,643 adult patients (aged ≥ 50 years) from 151 healthcare organizations (HCOs) within the TriNetX network were screened (Figure 1). During the study period, 71,524 patients were identified with serum zinc levels below 70 μg/ml, and 99,975 patients had zinc levels within the normal range. After applying the exclusion criteria, 39,048 patients remained in the zinc deficiency group and 65,379 in the control group. After 1:1 propensity score matching, 34,249 patients were included in each group. Propensity score density plots demonstrated a substantial imbalance between groups prior to matching, with limited overlap in score distributions (Figure 2). After matching, the two cohorts achieved excellent balance, with near-complete overlap of distributions and standardized mean differences < 0.1 across all covariates, confirming the adequacy of the matching procedure.

Prior to matching (Table 1), patients with zinc deficiency exhibited a higher prevalence of complex medical conditions, such as hypertension (33.9% vs. 27.4%), neoplasms (25.1% vs. 18.5%), and malnutrition (11.2% vs. 3.5%). These patterns suggest that zinc deficiency frequently co-occurs with broader systemic illnesses, reflecting an overall state of medical vulnerability rather than a standalone nutritional imbalance. Following 1:1 propensity score matching, the final cohorts were well balanced across demographic and clinical variables. The mean ages were nearly identical (61.1 ± 11.4 years vs. 60.9 ± 11.1 years), and sex distribution remained similar (34.7% vs. 33.2% male). Critical comorbidities relevant to cognitive risk, including hypertension (31.3% vs. 31.8 %), diabetes mellitus (16.0% vs. 16.1%), and obesity (20.6% vs. 21.6%), were closely aligned between groups. The use of benzodiazepines (31.2% vs. 32.1%) and antidepressants (24.1% vs. 24.5%) was nearly identical across the groups, reducing the likelihood that pharmacological confounding influenced the association between zinc deficiency and dementia risk.

**Table 1:** Baseline characteristics of patients before and after propensity score matching.

**Variables**	**Before matching**			**After matching**		
	**ZD group (*****n*** = **39,048)**	**Control group (*****n*** = **65,379)**	**SMD** ^†^	**ZD group (*****n*** = **34,249)**	**Control group (*****n*** = **34,249)**	**SMD** ^†^
**Patient characteristics**						
Age at index (years)	61.9 ± 11.6	59.3 ± 10.5	0.236	61.1 ± 11.4	60.9 ± 11.1	0.010
Male	14,236 (36.5%)	20,818 (31.8%)	0.097	11,883 (34.7%)	11,383 (33.2%)	0.031
BMI kg/m^2^	29.6 ± 9.0	30.6 ± 8.5	0.109	30.1 ± 9.0	30.1 ± 8.5	0.032
White	26,339 (67.5%)	45,817 (70.1%)	0.057	23,316 (68.1%)	22,901 (66.9%)	0.026
Unknown Race	5,426 (13.9%)	9,493 (14.5%)	0.018	4,882 (14.3%)	5,016 (14.6%)	0.011
Black or African American	4,701 (12.0%)	5,980 (9.1%)	0.094	3,837 (11.2%)	4,088 (11.9%)	0.023
Factors influencing health status and contact with health services	22,255 (57.0%)	32,220 (49.3%)	0.155	18,514 (54.1%)	18,912 (55.2%)	0.023
**Comorbidities**						
Essential (primary) hypertension	13,247 (33.9%)	17,945 (27.4%)	0.141	10,710 (31.3%)	10,889 (31.8%)	0.011
Dyslipidemia	9,899 (25.4%)	15,654 (23.9%)	0.033	8,262 (24.1%)	8,502 (24.8%)	0.016
Neoplasms	9,807 (25.1%)	12,098 (18.5%)	0.161	7,568 (22.1%)	7,820 (22.8%)	0.018
Overweight and obesity	8,081 (20.7%)	13,007 (19.9%)	0.020	7,069 (20.6%)	7,385 (21.6%)	0.023
Sleep disorders	7,127 (18.3%)	10,980 (16.8%)	0.038	6,055 (17.7%)	6,172 (18.0%)	0.009
Diabetes mellitus	7,064 (18.1%)	8,627 (13.2%)	0.135	5,465 (16.0%)	5,527 (16.1%)	0.005
Mood disorders	6,302 (16.1%)	8,384 (12.8%)	0.094	5,048 (14.7%)	5,188 (15.1%)	0.011
Vitamin D deficiency	5,473 (14.0%)	8,651 (13.2%)	0.023	4,657 (13.6%)	4,823 (14.1%)	0.014
Anemias	6,642 (17.0%)	5,563 (8.5%)	0.257	4,423 (12.9%)	4,495 (13.1%)	0.006
Ischemic heart diseases	5,073 (13.0%)	4,637 (7.1%)	0.197	3,475 (10.1%)	3,499 (10.2%)	0.002
Diseases of liver	5,104 (13.1%)	4,328 (6.6%)	0.218	3,435 (10.0%)	3,376 (9.9%)	0.006
Deficiency of other B group vitamins	2,797 (7.2%)	3,860 (5.9%)	0.051	2,314 (6.8%)	2,406 (7.0%)	0.011
Malnutrition	4,362 (11.2%)	2,274 (3.5%)	0.299	2,222 (6.5%)	2,152 (6.3%)	0.008
Nicotine dependence	2,866 (7.3%)	2,923 (4.5%)	0.122	2,061 (6.0%)	2,073 (6.1%)	0.001
Atrial fibrillation and flutter	3,030 (7.8%)	2,367 (3.6%)	0.179	1,907 (5.6%)	1,915 (5.6%)	0.001
COVID-19	1,922 (4.9%)	2,340 (3.6%)	0.067	1,539 (4.5%)	1,566 (4.6%)	0.004
**Chronic kidney disease (CKD)**						
CKD, stage 1	76 (0.2%)	76 (0.1%)	0.020	53 (0.2%)	55 (0.2%)	0.001
CKD, stage 2	434 (1.1%)	390 (0.6%)	0.056	291 (0.9%)	291 (0.9%)	< 0.001
CKD, stage 3	1,755 (4.5%)	1,437 (2.2%)	0.128	1,101 (3.2%)	1,141 (3.3%)	0.007
Cerebral infarction	1,124 (2.9%)	991 (1.5%)	0.093	760 (2.2%)	734 (2.1%)	0.005
Parathyroid disorders	385 (1.0%)	505 (0.8%)	0.023	310 (0.9%)	320 (0.9%)	0.003
Nontraumatic intracerebral hemorrhage	140 (0.4%)	113 (0.2%)	0.036	89 (0.3%)	88 (0.3%)	0.001
Nontraumatic subarachnoid hemorrhage	99 (0.3%)	76 (0.1%)	0.032	66 (0.2%)	59 (0.2%)	0.005
Alzheimer's disease	45 (0.1%)	21 (0.0%)	0.031	25 (0.1%)	19 (0.1%)	0.007
**Laboratory data**						
Hemoglobin≥12 mg/dL	22,291 (57.1%)	38,538 (58.9%)	0.038	19,745 (57.7%)	19,917 (58.2%)	0.010
Hemoglobin A1c ≥7%	3,418 (8.8%)	4,636 (7.1%)	0.062	2,780 (8.1%)	2,781 (8.1%)	< 0.001
Albumin g/dL (≥3.5 g/dL)	20,840 (53.4%)	33,226 (50.8%)	0.051	18,023 (52.6%)	18,629 (54.4%)	0.035
**Medications**						
Benzodiazepine derivative sedatives/hypnotics	13,559 (34.7%)	17,065 (26.1%)	0.188	10,690 (31.2%)	10,980 (32.1%)	0.018
Antidepressants	10,185 (26.1%)	13,569 (20.8%)	0.126	8,263 (24.1%)	8,407 (24.5%)	0.010
Antiepileptics	9,604 (24.6%)	12,532 (19.2%)	0.132	7,772 (22.7%)	7,937 (23.2%)	0.011
Antilipemic agents	9,055 (23.2%)	12,530 (19.2%)	0.099	7,301 (21.3%)	7,490 (21.9%)	0.013
Anticholinergics	4,498 (11.5%)	5,649 (8.6%)	0.096	3,562 (10.4%)	3,704 (10.8%)	0.013
Zinc supplements	1,462 (3.7%)	1,716 (2.6%)	0.064	1,131 (3.3%)	1,153 (3.4%)	0.004
GLP-1 analogs	1,106 (2.8%)	1,898 (2.9%)	0.004	994 (2.9%)	1,020 (3.0%)	0.004
SGLT2 inhibitors	661 (1.7%)	970 (1.5%)	0.017	524 (1.5%)	551 (1.6%)	0.006

ZD, zinc deficiency; BMI, body mass index; SMD, standardized mean differences; ^†^SMD values < 0.1 indicate adequate balance between groups; GLP-1, glucagon-like peptide-1; SGLT2, sodium-glucose co-transporter 2; CKD, chronic kidney disease.

### 3.2 Association between zinc deficiency and 3-year dementia risk

The primary analysis revealed a statistically significant association between zinc deficiency and an increased risk of new-onset dementia over a 3-year follow-up period (Table 2). Among patients with zinc deficiency, 336 (0.98%) developed dementia compared with 305 (0.89%) in the control group. After adjusting for potential confounders, zinc deficiency was associated with a 34% increased risk of dementia (adjusted HR 1.34, 95% CI 1.17–1.53, *p* < 0.001), and the proportional hazards assumption was confirmed using Schoenfeld residuals. To validate our analytical approach, we examined pneumonia as a positive control outcome, given the established relationship between zinc deficiency and immune dysfunction. As anticipated, zinc-deficient patients demonstrated substantially higher pneumonia rates (6.4% vs. 4.6%) with a robust 72% increase in risk (adjusted HR 1.72, 95% CI 1.63–1.81, *p* < 0.001). This finding strengthens confidence in our methodology and supports the biological plausibility of the role of zinc in health outcomes.

**Table 2:** Association between zinc deficiency and 3-year risk of dementia.

**Outcomes**	**ZD group^†^ (*n* = 34,249)**	**Control group^†^ (*n* = 34,249)**	**HR (95% CI)**	***p*-value**	**aHR**	***p*-value**
	**Events (%)**	**Events (%)**				
Dementia	336 (0.98%)	305 (0.89%)	1.20 (1.03–1.40)	0.023	1.34 (1.17–1.53)	< 0.001
Cognitive impairment	207 (0.60%)	222 (0.65%)	1.01 (0.84–1.22)	0.910	1.08 (0.92–1.28)	0.339
Pneumonia (positive control)	2,200 (6.4%)	1,583 (4.6%)	1.52 (1.42–1.62)	< 0.001	1.72 (1.63–1.81)	< 0.001

aHR, adjust hazard ratio; ZD, zinc deficiency; CI, confidence interval; ^†^The mean follow-up duration was 771 days in the ZD group and 834 days in the control group.

Interestingly, cognitive impairment without dementia showed no significant association with zinc status. The rates were nearly identical between groups (0.60% vs. 0.65%), resulting in a non-significant adjusted HR of 1.08 (95% CI 0.92–1.28, *p* = 0.339). This pattern suggests that zinc deficiency may specifically influence the progression to frank dementia rather than milder cognitive decline.

### 3.3 Temporal consistency: 5-year dementia risk

Extended follow-up to 5 years demonstrated remarkable consistency in our findings, reinforcing the temporal stability of the zinc-dementia association (Table 3). The cumulative incidence of dementia remained higher in the zinc deficiency group (1.32% vs. 1.26%), although the relative risk was somewhat attenuated compared to the 3-year analysis (adjusted HR 1.27, 95% CI 1.14–1.43, *p* < 0.001). This modest attenuation likely reflects the natural accumulation of dementia cases in the control group over extended observation periods, as other risk factors exert their influence over time. The pneumonia findings remained consistent across the longer timeframe, with zinc-deficient patients continuing to show an elevated risk (7.9% vs. 6.0%, adjusted HR 1.68, 95% CI 1.60–1.77, *p* < 0.001). Similarly, cognitive impairment without dementia continued to show no significant association (adjusted HR 1.09, 95% CI 0.94–1.25, *p* = 0.252), further supporting the specificity of zinc's relationship with dementia progression rather than general cognitive decline.

**Table 3:** Association between zinc deficiency and 5-year risk of dementia.

**Outcomes**	**ZD group^†^ (*n* = 34,249)**	**Control group^†^ (*n* = 34,249)**	**HR (95% CI)**	***p*-value**	**aHR**	***p*-value**
	**Events (%)**	**Events (%)**				
Dementia	453 (1.32%)	431 (1.26%)	1.16 (1.01–1.32)	0.032	1.27 (1.14–1.43)	< 0.001
Cognitive impairment	283 (0.83%)	306 (0.89%)	1.02 (0.864–1.19)	0.586	1.09 (0.94–1.25)	0.252
Pneumonia (positive control)	2,720 (7.9%)	2,042 (6.0%)	1.47 (1.39–1.56)	< 0.001	1.68 (1.60–1.77)	< 0.001

aHR, adjust hazard ratio; ZD, zinc deficiency; HR, hazard ratio; CI, confidence interval; ^†^The mean follow-up duration was 1,018 days in the ZD group and 1,182 days in the control group.

### 3.4 Sensitivity analysis

To evaluate the robustness of our findings and exclude potential COVID-19 pandemic confounding, we conducted a sensitivity analysis restricting the study period to January 1, 2010, through December 31, 2019 (Table 4). The pre-pandemic analysis included 20,888 patients per group after propensity score matching. Despite the small sample size, the association between zinc deficiency and dementia risk remained statistically significant. The adjusted HR of 1.28 (95% CI 1.07–1.55, *p* = 0.008) was remarkably consistent with our primary analysis, demonstrating that the zinc-dementia relationship was independent of pandemic-related healthcare disruptions. Pneumonia findings showed similar consistency, with zinc-deficient patients demonstrating a 67% increased risk (adjusted HR 1.67, 95% CI 1.55–1.80, *p* < 0.001). Notably, cognitive impairment without dementia became statistically significant in this pre-pandemic cohort (adjusted HR 1.38, 95% CI 1.11–1.72, *p* = 0.004), suggesting that pandemic factors may have influenced this relationship in our primary analysis.

**Table 4:** Sensitivity analysis: association between zinc deficiency and 3-year risk of dementia in pre-pandemic period (2010–2019).

**Outcomes**	**ZD group (*n* = 20,888)**	**Control group (*n* = 20,888)**	**HR (95% CI)**	***p*-value**	**aHR**	***p*-value**
	**Events (%)**	**Events (%)**				
Dementia	181 (0.87%)	175 (0.84%)	1.10 (0.89–1.35)	0.379	1.28 (1.07–1.55)	0.008
Cognitive impairment	134 (0.64%)	115 (0.55%)	1.24 (0.96–1.59)	0.096	1.38 (1.11–1.72)	0.004
Pneumonia (positive control)	1,264 (6.1%)	940 (4.5%)	1.43 (1.32–1.56)	< 0.001	1.67 (1.55–1.80)	< 0.001

ZD, zinc deficiency; Ahr, adjust hazard ratio; CI, confidence interval.

### 3.5 Dose-response relationship

The observed dose-response relationship across different levels of zinc deficiency provides additional support for a potential causal link (Table 5). Patients with mild-to-moderate zinc deficiency (50–70 μg/ml) showed a 26% increased risk of dementia (adjusted HR 1.26, 95% CI 1.10–1.46, *p* = 0.001), while those with severe deficiency (< 50 μg/ml) faced a substantially elevated 71% increased risk (adjusted HR 1.71, 95% CI 1.36–2.16, *p* < 0.001). This dose-response pattern was even more pronounced for pneumonia outcomes, where severe zinc deficiency conferred more than triple the risk compared to normal zinc levels (adjusted HR 3.12, 95% CI 2.87–3.40, *p* < 0.001). The progressive increase in risk with worsening zinc deficiency provides biological evidence that zinc status directly influences these health outcomes rather than serving as a surrogate marker for other unmeasured factors.

**Table 5:** Dose-response relationship across different levels of zinc deficiency at 3-y follow up.

**Outcomes**	**HR**		**aHR**	
	**HR (95% CI)**	***p*** **-values**	**HR (95% CI)**	***p*** **-values**
**Mild-to-moderate zinc deficiency (50–70** μ**g/ml) vs. control**				
Dementia	1.22 (1.04–1.43)	0.014	1.26 (1.10–1.46)	0.001
Cognitive impairment	1.08 (0.89–1.31)	0.454	1.11 (0.93–1.32)	0.249
Pneumonia (positive control)	1.29 (1.21–1.38)	< 0.001	1.44 (1.35–1.53)	< 0.001
**Severe zinc level (**<**50** μ**g/ml) vs. control**				
Dementia	1.34 (1.01–1.79)	0.045	1.71 (1.36–2.16)	< 0.001
Cognitive impairment	1.20 (0.80–1.79)	0.370	1.22 (0.88–1.67)	0.230
Pneumonia (positive control)	2.23 (1.99–2.49)	< 0.001	3.12 (2.87–3.40)	< 0.001

aHR, adjust hazard ratio; CI, confidence interval.

Cognitive impairment did not exhibit a significant dose-response relationship across the levels of zinc deficiency. However, given that cognitive impairment became statistically significant in our pre-pandemic sensitivity analysis, the absence of dose-response relationships for this outcome may reflect temporal confounding rather than true biological independence.

### 3.6 Subgroup analyses by sex and age

The association between zinc deficiency and the 3-year risk of dementia remained consistent across both male (aHR 1.23) and female (aHR 1.37) participants, with no significant interaction detected (*p* for interaction = 0.404) (Table 6). Similarly, cognitive impairment and pneumonia outcomes did not demonstrate any significant sex-based interaction. When analyzed by age, the association between zinc deficiency and dementia risk remained stable, with the interaction test showing no statistical difference (*p* = 0.453) (Table 7). Interestingly, the link between zinc deficiency and pneumonia was stronger in the younger age group (*p* for interaction = 0.013). Taken together, these subgroup analyses supported that the relationship between zinc deficiency and dementia risk is robust and not meaningfully influenced by age or sex.

**Table 6:** Subgroup analysis based on sex at 3-y follow up.

**Outcomes**	**Male**		**Female**		***p* for interaction**
	**aHR (95% CI)**	***p*** **-values**	**aHR (95% CI)**	***p*** **-values**	
Dementia	1.23 (1.02–1.49)	0.035	1.37 (1.16–1.62)	< 0.001	0.404
Cognitive impairment	1.07 (0.84–1.37)	0.586	1.13 (0.92–1.39)	0.256	0.740
Pneumonia (positive control)	1.82 (1.68–1.97)	< 0.001	1.65 (1.54–1.77)	< 0.001	0.072

aHR: adjust hazard ratio; CI: confidence interval.

**Table 7:** Subgroup analysis based on age at 3-y follow up.

**Outcomes**	**50–75 years**		>**75 years**		***p* for interaction**
	**aHR (95% CI)**	***p*** **-values**	**aHR (95% CI)**	***p*** **-values**	
Dementia	1.40 (1.12–1.75)	0.003	1.26 (1.10–1.47)	0.002	0.453
Cognitive impairment	1.19 (0.97–1.46)	0.096	0.96 (0.76–1.21)	0.712	0.175
Pneumonia (positive control)	1.78 (1.67–1.90)	< 0.001	1.55 (1.41–1.69)	< 0.001	0.013

aHR, adjust hazard ratio; CI, confidence interval.

### 3.7 Independent risk factors for dementia development

Multivariate analysis confirmed that older age and traditional cardiovascular risk factors, including cerebrovascular disease, diabetes, heart failure, and hypertension, were independently associated with an increased risk of dementia (Table 8). In contrast, obesity appeared to be protective, consistent with prior reports of an “obesity paradox” in dementia risk (38). Overall, these results align with established epidemiological evidence (39) and highlight the complex relationship between vascular health, aging, and dementia risk.

**Table 8:** Risk factors for new-onset dementia at 3-year follow up.

**Variable**	**aHR (95% CI)**	***p*-value**
Zinc deficiency vs. control group	1.34 (1.17, 1.53)	< 0.001
Male	1.04 (0.91, 1.20)	0.533
Essential (primary) hypertension	1.47 (1.24, 1.75)	< 0.001
Overweight and obesity	0.68 (0.56, 0.83)	< 0.001
Diabetes mellitus	1.52 (1.30, 1.79)	< 0.001
Cerebrovascular diseases	1.90 (1.59, 2.26)	< 0.001
Malnutrition	0.89 (0.70, 1.14)	0.357
Anemia, unspecified	1.09 (0.92, 1.30)	0.306
Other diseases of liver	1.07 (0.85, 1.35)	0.583
Ischemic heart diseases	1.12 (0.93, 1.34)	0.227
Age at index	1.10 (1.09, 1.11)	< 0.001
Dyslipidemia	0.96 (0.81, 1.13)	0.600
Chronic kidney disease (CKD)	1.19 (0.98, 1.44)	0.082
Heart failure	1.50 (1.23, 1.83)	< 0.001

aHR, adjust hazard ratio; CI, confidence interval.

## 4 Discussion

Our analysis of over 68,000 propensity-matched participants from a diverse healthcare network demonstrated that individuals with serum zinc levels below 70 μg/ml faced a significantly elevated risk of developing dementia compared to those with normal zinc status. The association remained consistent across multiple analytical approaches, including extended follow-up periods and sensitivity analyses, which excluded potential pandemic-related confounding factors. Importantly, we observed a clear dose-response relationship, with severe zinc deficiency conferring a substantially greater risk than mild-to-moderate deficiency, strengthening the argument for a causal relationship. The validation of our methodology using pneumonia as a positive control outcome further supports the biological plausibility of our findings.

Despite growing recognition of the importance of zinc in neurological health, studies specifically investigating zinc deficiency as a risk factor for dementia remain remarkably scarce in the literature. The vast majority of existing research has focused on cognitive impairment more broadly than formally diagnosed dementia, creating a significant gap in our understanding of the role of zinc in preventing the most severe forms of cognitive decline. The limited available evidence comes primarily from small-scale, cross-sectional studies that provide important insights but lack the power and temporal framework necessary to establish epidemiological associations. For instance, cross-sectional analyses of older adults have identified relationships between dietary zinc intake and cognitive performance (26, 27), while an observational study in industrial workers exposed to neurotoxins has demonstrated associations between reduced plasma zinc levels and cognitive dysfunction (22). Research on neurodegenerative disease models, including Huntington's disease, has shown that brain zinc deficiency can exacerbate cognitive decline (23). More recently, studies in populations with Parkinson's disease have suggested that serum zinc deficiency may be associated with an increased risk of conversion to dementia (24). However, these investigations have been fundamentally limited by design constraints. Most employed cross-sectional methodologies that cannot establish temporal relationships or causality, whereas others focused on highly specific disease populations that may not reflect the broader at-risk community.

In the current study, the consistent risk elevation across different analytical approaches suggests a meaningful clinical relationship. Zinc plays fundamental roles in synaptic transmission, neuronal development, and protection against oxidative stress (15–18). The dose-response relationship further supports these mechanistic pathways, indicating that the degree of zinc deficiency directly correlates with neurological risk. Clinically, these findings suggest that zinc status assessment should be considered in comprehensive geriatric evaluation, particularly for patients with malnutrition, chronic kidney disease, or gastrointestinal disorders. Unlike non-modifiable dementia risk factors, such as age or genetics, zinc deficiency can be addressed through dietary counseling and supplementation. The dose-response relationship indicates that prevention strategies should target optimization across the normal range, especially for vulnerable populations including institutionalized elderly and patients with chronic inflammatory conditions. Given the aging population and substantial dementia care burden, even modest reductions in dementia incidence through zinc optimization could yield significant healthcare cost savings, although implementation requires careful consideration of dosing, target populations, and cost-effectiveness.

Compared to previous studies, our cohort study offers several key methodological advantages. First, the use of a large multicenter cohort with over 68,000 propensity-matched participants provides substantially greater statistical power and generalizability. Second, the extended follow-up period (up to 3 and 5 years) allowed for a more accurate identification of incident dementia cases. Third, propensity score matching and detailed adjustment for a wide range of demographic, clinical, and medication variables minimized residual confounding. Finally, the inclusion of a positive control outcome (pneumonia) offers additional internal validation of our analytical approach. Together, these strengths address the key limitations of earlier investigations and support the robustness of our findings.

In our primary analysis, cognitive impairment did not demonstrate a statistically significant association with zinc status, suggesting that zinc deficiency may exert its effects more prominently on the progression to clinically diagnosed dementia than on earlier, milder forms of cognitive decline. However, in a sensitivity analysis restricted to the pre-pandemic period, this relationship was statistically significant, indicating that temporal factors may have influenced the observed associations. There are several plausible explanations for this observation. The COVID-19 pandemic has profoundly disrupted healthcare delivery, altering diagnostic practices, access to outpatient services, and patient care pathways. These disruptions may have disproportionately affected the identification and documentation of milder cognitive changes, such as cognitive impairment, while the diagnosis of more severe conditions, such as dementia—often recognized during acute care or hospitalization—was likely less impacted. Furthermore, the lower absolute incidence of cognitive impairment compared to dementia may have limited the statistical power to detect significant associations during the pandemic-affected period. Our sensitivity analysis strengthens the biological plausibility that zinc plays a protective role in cognitive health across a continuum, rather than being limited to late-stage neurodegeneration.

The inclusion of pneumonia as a positive control outcome served as a crucial methodological validation of our approach. Zinc deficiency is known to impair immune function, and previous research has demonstrated clear associations between zinc status and infection risk, particularly respiratory infections (40). The association observed between zinc deficiency and pneumonia risk, with effect sizes exceeding those for dementia, provides confidence in our analytical methods and data quality. The dose-response relationship for pneumonia was even more pronounced than that for dementia, with severe zinc deficiency conferring more than triple the risk compared to normal zinc levels. This pattern aligns with existing immunological research and validates our categorization of zinc deficiency severity. The consistency of the pneumonia findings across all analytical approaches strengthens the confidence that our observed dementia associations reflect true biological relationships rather than analytical artifacts or unmeasured confounding.

Several limitations of this study must be acknowledged when interpreting these findings. First, because only 0.2% of the screened TriNetX population had undergone zinc testing, the analyzed cohort represents a highly selected subgroup, likely enriched for individuals with underlying clinical concerns. This introduces potential selection bias and limits the external validity and generalizability of our findings. However, owing to the extremely large population size within the TriNetX network, it is not feasible to perform detailed baseline comparisons between tested and untested individuals. Second, misclassification cannot be completely excluded when diagnoses rely on administrative or hospital discharge coding. We focused on all-cause dementia as the primary outcome to maximize diagnostic accuracy and statistical power, because specific dementia subtypes (such as Alzheimer's disease and vascular dementia) are frequently underdiagnosed or misclassified in real-world electronic health records. Future studies with more detailed clinical and biomarker data are needed to explore subtype-specific associations. Third, the observational design precludes definitive causal inference, despite the temporal relationship and dose-response pattern observed. Residual confounding remains possible, as risk of dementia may correlate with unmeasured lifestyle factors (e.g., alcohol, smoking, physical activity), dietary patterns, education, socio-economic status, or underlying health conditions. Furthermore, our study was unable to account for certain important covariates, such as APOE4 genotype, physical activity, sleep quality, and mental health status, as these variables were not available in the TriNetX database. The absence of these factors may introduce residual confounding factors that should be considered when interpreting our findings. Fourth, we assessed zinc status at a single time point, and changes in zinc levels over time were not captured. Fifth, the relatively short follow-up period may have missed longer-term associations, although the consistency of findings across 3- and 5-year intervals suggests stable relationships. Finally, we did not incorporate death as a competing risk factor in our primary analysis. Although we acknowledge that competing risks from mortality, especially during the pandemic period, may influence dementia incidence estimates, our study focused on time-to-event analysis for new-onset dementia, consistent with most prior research using the TriNetX platform. Because analyses were conducted using the TriNetX platform's built-in algorithms, stratification by matched sets and adjustment for healthcare center variability were not feasible. This limitation may introduce residual confounding, although the standardized analytic framework has been widely applied in prior multicenter EHR-based studies.

## 5 Conclusion

This large-scale retrospective cohort study provides epidemiological evidence that zinc deficiency is independently associated with an increased risk of new-onset dementia. The observed dose-response relationship, temporal consistency, and methodological validation through positive control outcomes support the biological plausibility of this association. These findings support the consideration of zinc status assessment and optimization as potential components of dementia prevention strategies, particularly in high-risk populations. Future randomized controlled trials are warranted to establish causality and determine the optimal intervention protocols for cognitive protection through zinc supplementation.

## Data Availability

The raw data supporting the conclusions of this article will be made available by the authors, without undue reservation.
